# Fallen stock data: An essential source of information for quantitative knowledge of equine mortality in France

**DOI:** 10.1111/evj.12664

**Published:** 2017-02-13

**Authors:** J. Tapprest, E. Morignat, X. Dornier, M. Borey, P. Hendrikx, B. Ferry, D. Calavas, C. Sala

**Affiliations:** ^1^ Laboratory for Equine Diseases French Agency for Food, Environmental and Occupational Health and Safety (Anses) Goustranville France; ^2^ Epidemiology Unit French Agency for Food, Environmental and Occupational Health and Safety (Anses) Lyon, Cedex 07 France; ^3^ French horse and riding institute (IFCE) Paris France; ^4^ Scientific Directorate for Laboratories French Agency for Food, Environmental and Occupational Health and Safety (Anses) Lyon, Cedex 07 France

**Keywords:** horse, mortality, temporal variation, mortality ratio, epidemiological surveillance

## Abstract

**Background:**

Quantitative information about equine mortality is relatively scarce, yet it could be of great value for epidemiological purposes. In France, data from rendering plants are centralised in the Fallen Stock Data Interchange database (FSDI), managed by the French Ministry of Agriculture, while individual equine data are centralised in the French equine census database, SIRE, managed by the French horse and riding institute (IFCE).

**Objectives:**

To evaluate whether the combined use of the FSDI and SIRE databases can provide representative and accurate quantitative information on mortality for the French equine population and to propose enhancements of these databases to improve the quality of the resulting demographic information.

**Study design:**

Descriptive study.

**Methods:**

Mortality ratios for the French equine population were calculated per year between 2011 and 2014 and temporal variations in equine mortality modelled during the same period. Survival analyses were performed on a sample of equines traceable in both the FSDI and SIRE databases.

**Results:**

Estimates of the annual mortality ratios varied from 3.02 to 3.40% depending on the years. Survival rates of equines 2‐years‐old and over differed according to breed categories with the highest median age at death for the ponies. The weekly description of mortality highlighted marked seasonality of deaths whatever the category of equines. Modelling temporal variations in equine mortality also brought to light excess mortality.

**Main limitations:**

Insufficient traceability of equines between the two databases.

**Conclusion:**

The FSDI database provided an initial approach to equine death ratios on a national scale and an original description of temporal variations in mortality. Improvement in the traceability of equines between the FSDI and SIRE databases is needed to enable their combined use, providing a representative description of equine longevity and a more detailed description of temporal variations in mortality.

## Introduction

Equine mortality is both an economic and welfare issue. Several studies have focused on the causes of death in horses 1, 2, 3, 4, 5, 6, 7, 8, 9, 10, but information on quantitative mortality remains relatively scarce. Current published information on quantitative equine mortality in Europe is mainly derived from surveys, or databases of limited equine populations 5, 6, 7, 11, 12, despite recently reinforced EC regulations (Commission Regulation [EC] No. 504/2008 of 6 June 2008; Commission Regulation [EC] No. 1950/2006 of 13 December 2006; Commission Regulation [EU] No. 37/2010 of 22 December 2009 and Commission Regulation [EU] No. 262/2015 of 17 February 2015) that require the collection and centralisation of a minimum of information on horses. Even today, this information is often disseminated among various professional organisations and difficult to access for demographical or epidemiological purposes 13, 14.

In France, individual equine data are centralised in the French equine census database, SIRE, which is managed by the French horse and riding institute (IFCE). The SIRE database provides individual information (unique SIRE identification number, microchip number, date of birth, sex and breed) for 95% of French equines whose owners have complied with regulations 15, 16. Legally, owners are responsible for notifying the IFCE of any change during the life of the horse and in the event of death, have to return the animal's passport to the IFCE. However, owners do not systematically comply or take a long time in doing so, meaning that information in the SIRE database on the date of death of the horse is often unreliable, even when available.

In this context, rendering plants are the main source of quantitative equine mortality data, since all animal cadavers have to be collected by fallen stock companies (law of 31 December 1975). These data are centralised daily in the Fallen Stock Data Interchange database (FSDI), managed by the French Ministry of Agriculture 17, 18. A recent assessment of FSDI equine data 19 showed that their quality, if perfectible, is sufficient to provide detailed and representative descriptions of equine mortality.

No comprehensive quantitative information about equine mortality has yet been published. The objectives of this study were to evaluate whether the combined use of the comprehensive FSDI and centralised SIRE databases can provide representative and accurate quantitative information on mortality outside slaughterhouses for the French equine population and to propose enhancements of these databases to improve the quality of the resulting demographic information.

## Materials and methods

### Materials

#### Fallen stock data interchange (FSDI) database

The FSDI database contains records relating removal of cadavers from holdings where equines are kept (farms, livery yards, etc.) and veterinary premises: cases are registered during telephone calls, or online requests for the removal of one or more dead animals. Thereafter, data collected for each visit are the date and time of the removal request, date of removal, identification, address and postcode of the holding, number of animals collected and their age/breed category, individual identification number and an estimation of global cadaver weight. The FSDI database does not record individual information on equines, such as date of birth or sex. This information can be found in the SIRE database for animals whose individual identification numbers are registered in the FSDI database.

To explore equine mortality data, we selected the 139,821 visits registered in the FSDI base from 1 January 2011 to 31 December 2014, since records before 2011 were not considered comprehensive. For 95 visits, the time between the removal request and removal exceeded 8 days. Such a delay was considered erroneous due to the regulatory obligation to remove cadavers within 48 h on business days, so the corresponding animals were also excluded. In 1.3% of the visits, the number of animals collected was not filled in, yet the estimated weight was not null. The number of animals collected was thus set to one, as most of the documented visits (99.3%) corresponded to the removal of a single animal. Finally, 139,726 visits corresponding to 141,008 dead animals were kept for analyses.

The date of death was estimated as accurately as possible by using the date of removal request when available, or the date of removal visit otherwise (1.5%). Age/breed categories are used by fallen stock companies for billing the owner of the dead equine, with a specific billing rate for each category. Animals were thus classified into six age/breed categories: stillbirth and foal (animals <1‐year‐old) or yearling (≥1‐year‐old and <2 years old) and adult animals (≥2 years old) classified by breed: pony, donkey, draught horse and saddle horse (i.e. any horse breed except draught horse breeds). Recent assessment of FSDI equine data 19 showed an unclear delineation of the age categories for young animals, with an overlap between categories (stillbirth and foal and yearling) and these were grouped into a single young animal category for the purpose of this study.

#### Information from the SIRE database

The estimated size of the live equine population based on SIRE data is biased because owners rarely notify SIRE managers of the death of their animals (only around 30% of deaths are registered). An estimation of the population size is nevertheless calculated annually by the IFCE using the number of animals identified by microchip in each birth cohort and an estimation of the number of deaths each year in each cohort 15, 20, 21. This estimate is then refined based on additional data sources such as customs, the National Federation of Horse Racing, the French Equestrian Federation and regional surveys.

In order to evaluate the survival of equines by breed and sex, we extracted from the SIRE database individual data (exact breed, sex and date of birth) for a subset of 18,884 animals for which the identification number registered in the FSDI database was traceable in the SIRE database. We additionally excluded 1291 animals under 2 years old because young animals were underrepresented in the subset of animals with a correct identification number in the FSDI database 19 and an estimate of survival from birth would therefore be biased. For 7258 of the remaining animals, the day and month of birth was unknown, but an estimate of the year of birth was provided. This was due to a delay in their identification, such that the exact age at the date of identification was unknown (Table 1). As the foaling season is short and focused around April, we set the day of birth for all these animals at 15 April to calculate an age at death. Survival by sex and breed was studied only for animals 4 years old and over to prevent potential bias due to imbalance between entire males and geldings in the young equine population. In young populations, only entire males are represented the first year, but the number of geldings gradually increases through castration up to the age of 4 years, when the proportion of entire males and geldings becomes stable.

**Table 1:** Details of animals with ID traceable in the SIRE database, 2011–2014 period, France

SIRE categories	Number of animals with valid ID	Animals ≥2 years old		Animals ≥4 years old						
		Number ≥2 years	Number (proportion)^a^ with birth date known	Number ≥4 years	Number (proportion)^b^ per sex			Number (proportion)^c^ per sex with birth date known		
					Female	Gelding	Male	Female	Gelding	Male
Donkey	1015	967	93 (9.6)	901	560 (62.2)	145 (16.1)	196 (21.7)	45 (8.0)	3 (2.1)	22 (11.2)
Draught horse	1418	1187	947 (79.8)	1020	812 (79.6)	61 (6)	147 (14.4)	638 (78.6)	23 (37.7)	132 (89.8)
Pony	4435	4329	1024 (23.7)	4188	2307 (55.1)	1468 (35)	413 (9.9)	533 (23.1)	201 (13.7)	211 (51.1)
Saddle horse	12,016	11,110	8271 (74.4)	10,197	5558 (54.5)	3400 (33.3)	1239 (12.2)	4124 (74.2)	2203 (64.8)	1077 (86.9)
Total	18,884	17,593	10,335 (58.7)	16,306	9237 (56.7)	5074 (31.1)	1995 (12.2)	5340 (57.8)	2430 (47.9)	1442 (72.3)

a Number of animals ≥2 years old with date birth known/number of animal ≥2 years old.

b Number of animal ≥4 years old, per sex/number of animals ≥4 years old.

c Number of animals ≥4 years old with date birth known/number of animal ≥4 years old, per sex.

### Methods

#### Estimation of global mortality ratios

Estimates of annual mortality ratios (per year between 2011 and 2014) were calculated by dividing the number of dead animals collected per year by the estimated number of live equines in France.

#### Modelling temporal variations in equine mortality

Temporal variation in equine mortality was assessed for adult breed categories, i.e. saddle horse (72,237 animals), draught horse (7582 animals), pony (34,259 animals) or donkey (10,730 animals) and for the young animal category (16,200 animals).

Generalised additive mixed models (GAMMs) were used to model the number of equine deaths Y per week for each breed category, taking into account seasonality, trend and short‐term autocorrelations with the following model:

$$Y_{i}=f_{1}\left({\mathrm{week}}_{i}\right)+f_{2}\left({\mathrm{time}}_{i}\right)+e_{i}$$

where *Y*_*i*_ is the number of dead animals, week_*i*_ the week in the year (1,…52), time an index of time (1,…,208) and e_*i*_ = φ e_*i*−1_ + ε_*i*_, with ε_*i*_ the stochastic error normally distributed N(0,σ^2^) and φ the autocorrelation parameter, f_1_ a cyclic cubic regression spline with the value and first two derivatives matching at year ends and f_2_ a cubic regression spline 22. Model adequacy was checked by examination of residual plots. Statistical tests were performed to assess the significance of the trend and the seasonal component in the model 23.

Analyses were performed using R software packages ‘mgcv’ and ‘nlme’.

#### Survival analysis

The mean of the age at death and distribution of the year of birth (Supplementary Items 1 and 2) differed significantly between animals with and without a known birth date. Therefore, we excluded all animals without a complete date of birth from survival analyses. The exclusion of animals without an exact date of birth varied in breed categories: 90% of donkeys; 76% of ponies; 26% of saddle horses and 20% of draught horses (Table 1). Exclusion also varied with sex, entire males being less affected for all breeds (Table 1). Survival analyses stratified by breed were conducted on a subset of 10,335 equines, while 9212 animals remained for the survival analyses by breed and sex (Table 1). Given the low number of donkeys, no stratified survival analysis by sex was performed for this group (Table 1). Survival analyses were performed using the R software package ‘survival’. The survival curves were obtained from Kaplan–Meier estimates 24 and compared using a log rank test.

## Results

### Mortality ratio

We estimated that, over the 4 years of the study, 3.17% of equines from the live population were removed by fallen stock companies. The mortality ratio varied significantly depending on the year (Table 2), with a higher mortality during 2012 and 2013, than the first and last years of the study period.

**Table 2:** Annual number of live and dead French equines and mortality ratio with 95% confidence intervals (CI) in 2011–2014

Year	Number of equines registered in the FSDI database	Number of live equines estimated from the SIRE database	Mortality ratio (%)		
			Mean	Lower CI	Upper CI
2011	33,953	1,114,000	3.05	3.02	3.08
2012	35,817	1,118,000	3.20	3.17	3.24
2013	37,456	1,112,000	3.37	3.33	3.40
2014	33,782	1,106,000	3.05	3.02	3.09
All	141,008	4,450,000	3.17	3.15	3.19

### Temporal variations in mortality

**Figure 1:**
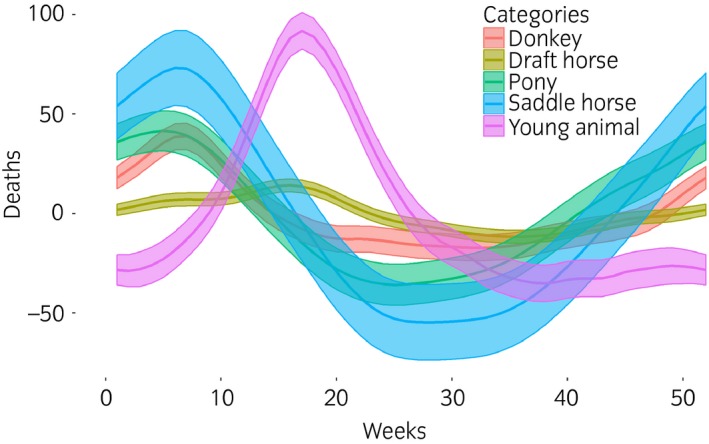
Annual seasonality and 95% confidence interval of French equine mortality estimated from 2011 to 2014 using Generalised Additive Mixed Models.

**Figure 2:**
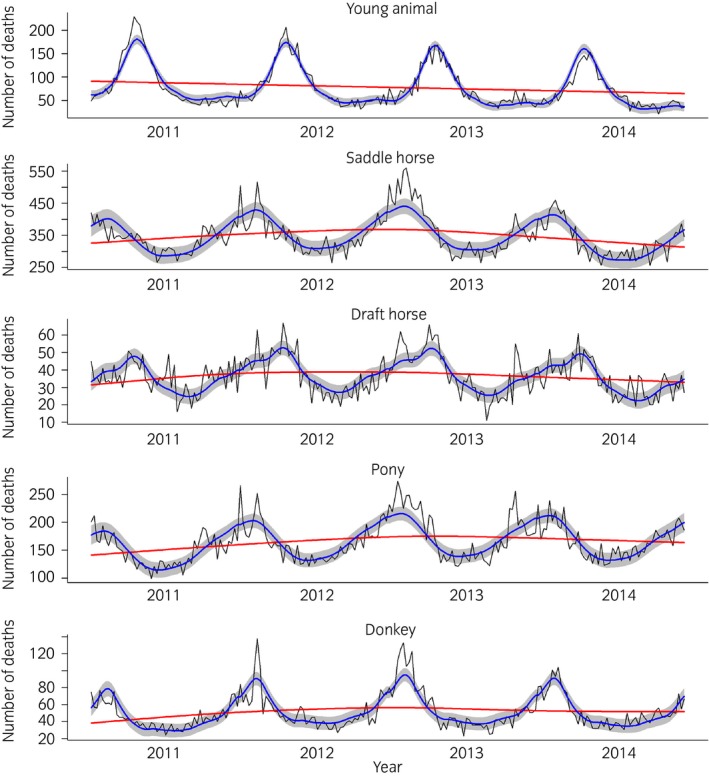
Temporal variation of French equine mortality from 2011 to 2014 per age‐breed category; observed value in black, predicted values of the model in blue and the trend in red; the grey area corresponds to the 95% confidence interval of the predicted value.

The results of temporal modelling are presented in Figures 1 and 2. In all models, examination of residual plots did not reveal any substantial deviation from normal distribution. In all models, both the trend and seasonal component were statistically significant (P<10^−6^ and <0.006, respectively). For young animals*,* mortality peaked in late April to early May and a minimum was reached from September to January, while the global trend decreased linearly from 2011 to 2014 (Figs 1, 2).

The temporal variation in the mortality of adults belonging to the saddle horse, pony or donkey categories was similar, with a peak in early February and a minimum in late June (Fig 1), while the seasonality of draught horse mortality differed, the main peak occurring later in late April and a minimum in September (Fig 1). The trend was not linear for the adult categories, but increased during the first 2 years before decreasing (Fig 2).

Finally, the observed values exceeded the values predicted by the model during the winters of 2011–2012 and 2012–2013 for the adult categories and during spring 2011 and 2012 for young animals.

### Survival analyses

**Figure 3:**
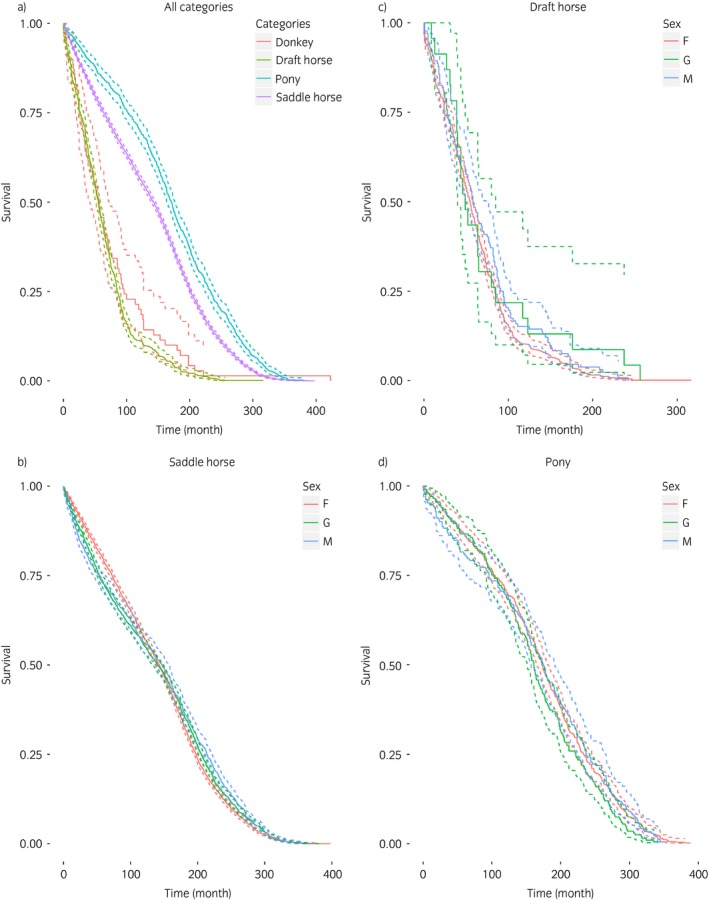
Survival curves (solid lines) and 95% confidence intervals (dashed lines) per breed for 10,335 French equines ≥2 years old a) and per breed and sex (F: female, G: gelding, M: male) for animals ≥4 years old (7404 saddle horses b), 793 draught horses c) and 945 ponies d)).

The survival curves are presented in Figure 3. Survival rates at 10 and 20 years and median ages at death are available in the additional material (Supplementary Items 3 and 4). The global log rank test demonstrated significant differences between breed categories in survival for adults over 2 years old (P<0.0001). With a median age at death of 16.9 years, the pony category had the highest survival rate, followed by saddle horse with a median age at death of 14.3 years. The survival rates for draught horse and donkey categories were the shortest, with median ages at death of 8.3 and 8.4 years, respectively.

No significant differences in survival rates for animals over 4 years old according to sex were found between any breed categories (log rank tests, P = 0.09).

## Discussion

The current study on mortality, which included natural deaths and euthanasia, but excluded slaughtering (around 30% of equine mortality in France), was based on an exhaustive source of mortality data 20. Estimates of the annual overall mortality ratios for the French equine population lay within the 3.02–3.40% interval. Estimates of equine mortality ratio vary markedly with the definition of mortality rates, type of samples involved and diversity of situations. In France, Leblond *et al*.^7^ observed an annual death rate of 2.47% in a retrospective study on 448 insured horses, while the National Animal Health Monitoring System (NAHMS, USA) published an annual death rate of 1.8% for animals over 30 days old based on a large sample of equines from 28 states 25. In Germany, an annual death rate of 2.2% has been reported in racehorses 12. In a large population of insured Swedish horses, Egenvall *et al*.^5^ estimated an incidence‐based mortality rate of 415 deaths per 10,000 horse‐years. In our study, all age categories were included, encompassing animals under 12 months old for which the mortality ratio is higher than in the adult population 26, 27. This could explain the higher mortality ratio we calculated compared with previous studies on populations excluding very young animals. Our estimates are based on comprehensive data covering the whole French equine population and recorded over four successive years. They should be more representative of mortality for the general population, even if some animals (stillbirths or very young foals) could potentially escape the rendering plant through illegal burial. An additional potential bias in our study could be due to the uncertainty of the estimated size of the live equine population.

A temporal description of equine mortality revealed marked seasonality, whatever the category of animals. For young animals, the mortality peak in the spring is superimposed on the foaling season (increase in the population at risk). The young animal category was nonetheless too broad and did not provide for an accurate description of the specific seasonality characteristics for neonates, foals and yearlings. For saddle horse, pony and donkey categories, the mortality peak in winter could presumably be related to adverse weather conditions and/or zootechnical factors (such as inadequate winter housing or increased infection pressure when animals are housed) as suggested in studies on the seasonality of cattle mortality 28, 29. For the draught horse category, with a different pattern of mortality, seasonality could reflect specific zootechnical factors such as extensive farming methods, more problems during foaling than in light breeds, metabolic disorders during the intensive growth of grass in early spring, etc. 30, 31. These factors can only be hypothesised and further investigations into their possible impact on mortality are necessary.

The temporal description showed a global trend over the 4 years studied. A linear decrease in the deaths of young equines from 2011 to 2014 was apparent and can be partly explained by the concomitant decrease in births in France (58,027 births in 2011 vs. 40,720 births in 2014) 20. For all adult categories, an increase in deaths was noted over the first 2 years, followed by a decrease. This trend follows changes in the equine population from 2011 to 2014 20, caused by historical variations in births. The weekly description of mortality also highlighted apparent excess mortality during the winters of 2011–2012 and 2012–2013. Insofar as no unusual health events had affected the French horse industry during these periods, the causes of this excess mortality are unknown and appear difficult to explore retrospectively. Nevertheless, the possibility of an objectification of abnormal peaks through the modelling of FSDI data strongly suggests its potential for the surveillance of equine mortality.

Lastly, a combination of FSDI and SIRE data allowed us to explore survival by breed and sex for animals over a certain age. The survival analysis by breed category was limited to animals that had already reached the age of 2 years and did not investigate the mortality of foals or yearlings. The uncertainty about the year of birth of the animals whose date of birth was not known fully justifies their exclusion but reduces the sample size and potentially biases the subset used for the survival analyses. Another limit of the survival analyses is that the subset used was not fully representative of the general population of adults registered in the FSDI database, having a higher percentage of saddle horses and a lower percentage of donkey and pony categories. Moreover, in the draught horse group, mares were largely dominant. Among draught horses, many entire males are intended for slaughter, which probably explains why these animals are no longer present in a population of animals over 2 years old. This is consistent with other survival analyses, that were based on the deaths registered in the SIRE base (natural deaths, euthanasia and slaughter) and in particular with the early and rapid decrease in the survival curve during the first 3 years for a population of draught horses intended for slaughter 15, 21.

Survival analyses showed that survival differed significantly according to the breed category. The longevity of ponies documented in our study has also been found in other studies in the USA and Sweden 6, 32. Different reasons have been advanced to explain this fact, such as the greater hardiness of ponies, a less intense workload 6 and a greater capability for responding to and repairing tissue damage 32. The shorter lifespan of draught horses compared with other horse breeds is coherent with the results of a previous study by Dornier 15. Various reasons for this shorter lifespan have been suggested in a study of insured French horses, including an increased risk during foaling for this breed group due to the size of the foal, or the lower value and rougher life style of these animals 7. In fact, better knowledge of the underlying population of living draught horses would help in interpreting this result. Finally, the very low number of donkeys (93 animals) limited exploration of their survival. Other studies are needed to clarify the reasons for such big differences in longevity between breeds. Indeed, from an economic, ethical and animal welfare perspective, it would be worthwhile to further investigate the reasons underlying equine longevity.

The survival of animals aged 4 years old and over did not reveal differences in longevity between geldings, entire males and mares. For draught horse and pony categories, the limited number of animals did not allow us to draw conclusions, but the number of animals included would have been sufficient to reveal any sex‐related survival differences for saddle horses. Contrary to the results of our study, Egenvall *et al*.^5^ found that between the ages of 4 and 15 years, geldings had a higher risk of death than mares and stallions. They suggested that geldings were much more likely to be subjected to euthanasia as a result of nonlife‐threatening problems because they cannot be used for breeding, whereas mares with the same condition might be kept as brood mares. Other studies found a longer lifespan for mares than for entire males 33, 34.

One possible reason for the discrepancy between our results and these studies might be that our study population does not focus on explicit populations such as insured horses, Thoroughbreds, etc., which can present specificities regarding the overall and specific risk of death of stallions, geldings and mares. Indeed, our results are an estimate of the average figures for the global French equine population.

## Conclusion

The FSDI database has proven to be a valuable source of data for studying mortality in the French equine population. It provided an initial approach to equine death ratios on a national scale and an original description of temporal variations in mortality based on a large population of dead equines (141,008). However, the combined use of the FSDI and SIRE databases was limited by the small proportion of equines (18,884) for which the identification number was both registered in the FSDI database and traceable in the SIRE database. Therefore, results of the survival analyses should only be considered as a preliminary approach to the longevity of French equines. The systematic registration of identification numbers is the most crucial improvement required of the FSDI database. Indeed, such an improvement would enable comprehensive interoperability and synergistic use of the FSDI and SIRE databases. In fact, this would provide a representative description of longevity, a detailed temporal description and modelling of equine mortality by smaller age groups, sex and smaller breed groups and near real time monitoring of equine mortality.

Moreover, such an improvement in the FSDI database would enhance the estimation of the live equine population by more accurate knowledge of dead animals currently registered in the SIRE database, because owners do not systematically declare deaths to the SIRE database managers. Furthermore, cadaver disposal data were used to provide a temporal description, model deaths and objectify excess mortality, thus suggesting its potential interest for equine health surveillance as already documented for human and cattle mortality 18, 35, 36. With this goal in mind, useful data, in particular the type of death (natural or euthanasia), could also be collected in the future from owners on a voluntary basis when they request online removal of their dead equine. Indeed, attitudes regarding the practice of euthanasia can vary greatly between breeds, between countries and over time and without any link to an outbreak. They do, however, depend on other factors such as ethical guidelines, economic factors and changes in regulations with regard to slaughterhouse admission, etc. It would also be useful to collect data on the probable causes of death. Slaughtering data should also be taken into account, as variations in these data could lead to misleading interpretations of certain excess mortality figures brought to light through the FSDI database.

Even if not used for detection purposes, equine mortality monitoring could represent an efficient tool for evaluating the impact and evolution of identified health events. It could therefore help in decision‐making and assessing the efficiency of control measures. However, unlike for cattle, the use of the FSDI database for equine mortality surveillance is limited by incomplete knowledge of the underlying population of living equines. Further work is therefore needed to evaluate the feasibility of implementing syndromic surveillance for the horse industry based on equine mortality.

## Authors’ declaration of interests

No competing interests have been declared.

## Ethical animal research

Research ethics committee oversight not currently required by this journal: retrospective study of official databases. Explicit owner informed consent for inclusion of animals in this study was not stated.

## Sources of funding

None.

## Authorship

J. Tapprest, M. Borey, P. Hendrikx and C. Sala contributed to the conception and design of the study; X. Dornier, E. Morignat and C. Sala contributed to the acquisition of data; J. Tapprest, E. Morignat, B. Ferry, C. Sala and D. Calavas contributed to the analysis and interpretation of data; the manuscript was drafted by J. Tapprest and C. Sala and critically revised by E. Morignat, X. Dornier, M. Borey, D. Calavas, P. Hendrikx and B. Ferry; all authors contributed to final approval of the version to be submitted.

## Supporting information


**Supplementary Item 1:** Mean age at death 17,593 French equines ≥2 years old.

**Supplementary Item 2:** Year of birth of 17,593 French equines ≥2 years old.

**Supplementary Item 3:** Survival analyses for 10,335 French equines ≥2 years old.

**Supplementary Item 4:** Survival analyses for French equines ≥4 years old.
