# Supplementary material for: Fallen stock data: An essential source of information for quantitative knowledge of equine mortality in France
Source: Equine Vet J. 2017 Feb 13;49(5):596–602. doi: 10.1111/evj.12664 (PMC5573972; doi:10.1111/evj.12664)
Supplement: Supplementary file 1 — Supplementary Item 1: Mean age at death 17,593 French equines ≥2 years old. [file EVJ-49-596-s001.pdf]

Equine Veterinary Journal

Supplementary Item 1: Mean age at death and 95% confidence interval for 17,593 French equines ≥2 years old with ID traceable in the SIRE database.

SIRE categories	Mean age at death (months) and 95% confidence interval of animals	
	With birth date known (N = 10,335)	With birth date unknown (N = 7,258)
**Donkey**	101.2 [86.6-115.9]	176.7 [170.9-182.6]
**Draught horse**	99.8 [96.4-103.2]	208.4 [197.3-219.4]
**Pony**	202.5 [196.6-208.5]	251.3 [248.2-254.5]
**Saddle horse**	171.7 [169.6-173.7]	256.0 [252.8-259.2]
