# Supplementary material for: Fallen stock data: An essential source of information for quantitative knowledge of equine mortality in France
Source: Equine Vet J. 2017 Feb 13;49(5):596–602. doi: 10.1111/evj.12664 (PMC5573972; doi:10.1111/evj.12664)
Supplement: Supplementary file 2 — Supplementary Item 2: Year of birth of 17,593 French equines ≥2 years old. [file EVJ-49-596-s002.pdf]

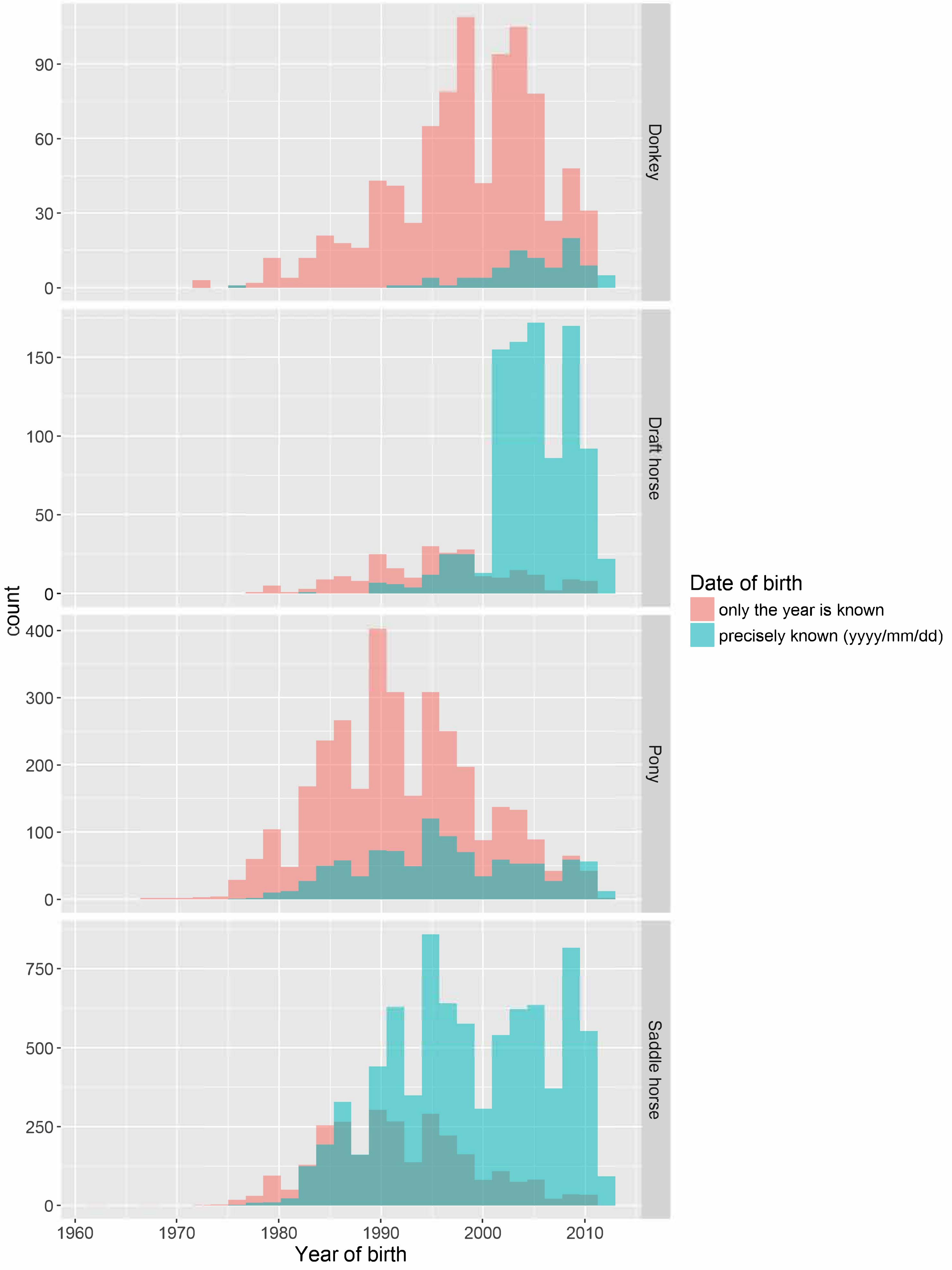

Supplementary Item 2: Distribution of the year of birth of 17,593 French equines ≥2 years old per breed, according to knowledge of the precise date of birth.
