# Supplementary material for: Fallen stock data: An essential source of information for quantitative knowledge of equine mortality in France
Source: Equine Vet J. 2017 Feb 13;49(5):596–602. doi: 10.1111/evj.12664 (PMC5573972; doi:10.1111/evj.12664)
Supplement: Supplementary file 3 — Supplementary Item 3: Survival analyses for 10,335 French equines ≥2 years old. [file EVJ-49-596-s003.pdf]

Equine Veterinary Journal

Supplementary Item 3: Results of survival analyses for 10,335 French equines ≥2 years old per category: median age at death and survival rates at 10 and 20 years.

SIRE categories	Number of equines	Median age at death (years)	Survival rate (%) and 95% confidence interval at	
			10 years	20 years
**Donkey**	93	8.4	18.3 [11.9-28.1]	2.2 [0.6-8.5]
**Draught horse**	947	8.3	14.5 [12.5-17.0]	1.3 [0.7-2.2]
**Pony**	1,024	16.9	70.5 [67.8-73.4]	27.9 [25.3-30.8]
**Saddle horse**	8,271	14.3	57.8 [56.8-58.9]	17.7 [16.9-18.6]
