# Supplementary material for: Fallen stock data: An essential source of information for quantitative knowledge of equine mortality in France
Source: Equine Vet J. 2017 Feb 13;49(5):596–602. doi: 10.1111/evj.12664 (PMC5573972; doi:10.1111/evj.12664)
Supplement: Supplementary file 4 — Supplementary Item 4: Survival analyses for French equines ≥4 years old. [file EVJ-49-596-s004.pdf]

Equine Veterinary Journal

Supplementary Item 4: Results of survival analyses for French equines ≥4 years old per category and sex: median age at death and survival rates at 10 and 20 years.

SIRE categories	Sex	Number of Equines	Median age at death (years)	Survival rate (%) and 95% confidence interval at	
				10 years	20 years
**Draught horse**	**Female**	638	9.2	10.3 [8.2-12.9]	0.5 [0.2-1.44]
	**Gelding**	23	10.3	17.4 [7.1-42.4]	4.4 [0.6-29.6]
	**Male**	132	9.8	15.2 [10.1-22.7]	0.8 [0.1-5.3]
**Pony**	**Female**	533	18.3	70.9 [67.2-74.9]	22.7 [19.4-26.6]
	**Gelding**	201	17.4	69.7 [63.6-76.3]	18.9 [14.2-25.2]
	**Male**	211	18	69.7 [63.7-76.2]	24.2 [19.0-30.7]
**Saddle horse**	**Female**	4,124	15.7	57.5 [56.0-59.0]	12.1 [11.1-13.1]
	**Gelding**	2,203	15.5	55.4 [53.3-57.5]	13.7 [12.3-15.2]
	**Male**	1,077	15.7	56.2 [53.3-59.2]	15.9 [13.9-18.2]
